# Per-service supervised learning for identifying desired WoT apps from user requests in natural language

**DOI:** 10.1371/journal.pone.0187955

**Published:** 2017-11-17

**Authors:** Young Yoon

**Affiliations:** Department of Computer Engineering, Hongik University, Seoul, South Korea; Santa Clara University, UNITED STATES

## Abstract

Web of Things (WoT) platforms are growing fast so as the needs for composing WoT apps more easily and efficiently. We have recently commenced the campaign to develop an interface where users can issue requests for WoT apps entirely in natural language. This requires an effort to build a system that can learn to identify relevant WoT functions that fulfill user’s requests. In our preceding work, we trained a supervised learning system with thousands of publicly-available IFTTT app recipes based on conditional random fields (CRF). However, the sub-par accuracy and excessive training time motivated us to devise a better approach. In this paper, we present a novel solution that creates a separate learning engine for each trigger service. With this approach, parallel and incremental learning becomes possible. For inference, our system first identifies the most relevant trigger service for a given user request by using an information retrieval technique. Then, the learning engine associated with the trigger service predicts the most likely pair of trigger and action functions. We expect that such two-phase inference method given parallel learning engines would improve the accuracy of identifying related WoT functions. We verify our new solution through the empirical evaluation with training and test sets sampled from a pool of refined IFTTT app recipes. We also meticulously analyze the characteristics of the recipes to find future research directions.

## Introduction

Web of Thing (WoT) platforms such as IFTTT and Zaiper are on the rise. They provide intuitive graphical programming interface that supports users to easily and flexibly compose applications with various things connected on the Web [1, 2]. For instance, a user can request the WoT platforms to store newly appeared personal Foursquare point of interest (POI) logs to Google Calendar. This logic can be defined simply in a production rule, i.e., in a if-this-then-that fashion. The user first picks Foursquare’s POI function and configures it in the conditional clause (if-clause). Then the user adds Google’s calendar event addition function in the action clause (then-clause). The function that triggers an action is called *trigger function*. The function that is fired by the trigger function is referred to as *action function*. Trigger function specifies subscriptions to interested events (e.g., POI log appearance). An *action* function specifies the event-driven actuation (e.g., Philips Hue light bulb is turned on automatically at sunset). We defined *service* as a provider of these functions (e.g. Google Calendar, Foursquare and Philips Hue). We refer to the complete specification of a WoT app as a *recipe*.

For WoT service developers, WoT platforms offer a REST endpoint to which trigger events can be forwarded. The developers can also share the REST endpoint of actions with the WoT platforms so that it can execute the function on behalf of the user whenever the desired trigger condition is satisfied. The ecosystem around the WoT platforms is growing fast. For example, more than 300 services are connected to IFTTT, and they are providing thousands of trigger and action functions.

However, it is not clear whether the current graphical mash-up interface can appeal to broader user groups. For instance, Yahoo Pipes used to offer a web-based graphical programming interface for implementing pipelines of functions for filtering various third-party RSS feeds [3]. Despite its visually intuitive interface, composing the pipelines seemed to be cumbersome and difficult for many users, and it eventually ceased the operation in 2015.

This usability issue has prompted us to develop a novel interface that lets users to specify desired WoT app recipes entirely in natural language. We first collaborated with a research team at Samsung Electronics to implement a conceptual demo application in a voice-based personal assistant system [4]. This application converts voice queries to natural language text and identifies if-clause and then-clause. Then this application automatically activates a composition of relevant WoT functions on IFTTT platform. However, this application fell short in correctly identifying the true intention behind the requests that are oftentimes ambiguous and irregular. For instance, suppose a user issues a request such as “*If any breaking news in sports gets published*, *notify me*“. In such a request, the exact news source is not specified, and the request can be expressed quite differently such as “*Let me know whenever there is a breaking sports news*”. It was also difficult for our system to recognize which parts of the sentence relates to a desired trigger or an action.

Such shortcoming of our first work prompted us to investigate the feasibility of devising an engine that can *learn* what triggers and actions are actually asked for in the requests issued in natural language. We generated training sets out of publicly available WoT app recipes on IFTTT and developed a supervised learning method to identify the semantics of the user requests [5]. We employed a learning method [6, 7] based on conditional random fields (CRF) that has been effective in natural language processing (NLP) operations such as part-of-speech (POS) tagging and named entity recognition. We took POS-tagged sequence of tokens as a feature of a user request and modeled its relationship with a combination of trigger and action functions that we refer to as a *main act*. After the modeling, our learning engine predicts the most relevant main act for a given user request. We also adapted the named entity recognition approach by training our engines to learn the association of each token in a recipe with either a trigger function or an action function.

To the best of our knowledge, the aforementioned series of research work was the first-ever attempt to automatically compose an WoT app from thousands of in-production WoT functions for any given user requests that are expressed entirely in natural language. However, this ambitious initial effort fell short in accurately identifying correct WoT functions even with the CRF-based modeling [5]. Specifically, the highest accuracy we could achieve was only 51%. Moreover, the training time was excessively long, which made our solution impractical. In our preceding work, we tried to build a single unified model while WoT app recipes can be generated out of a pool of highly diverse functions. Therefore, our learning engine had to be optimized on a large number of parameters, which made the learning procedure impractical.

In this paper, we take a different approach by decompose the pool of WoT functions by trigger services. We conduct CRF-based modeling separately on a training set associated with each trigger service. For inference, the most relevant trigger service for a user request is first retrieved. Then, related WoT functions are predicted based on the CRF-based model associated with the trigger service. Through this new approach that generates parallel learning engines, we expect to improve the accuracy. Also, the separate learning on a smaller set of training data can increase the learning efficiency.

In the rest of this paper we present our contributions in the following structure: (1) we introduce the previous learning procedure which is the foundation for our new solution; (2) we present the new learning and testing approach; (3) we discuss results from the empirical evaluation results and the characteristics of the training and the test data; (4) we discuss potential future research directions; and (5) we put our work in the context of personal assistants, natural language processing (NLP) and application composition research works.

## The basic learning framework

We first reiterate the overall learning framework developed in [4, 5]. As mentioned earlier, these works provide the foundation of our new approach.

We first collect WoT app recipes (called *recipes* for brevity) that are publicly available on IFTTT. We collect the recipes in a non-invasive manner and abided by the standard rules for protecting personal information. In the previous work, we randomly sampled up to 9,000 recipes and carried out the operations to refine the data as follows. We only accepted recipes that were expressed in English. We excluded recipes that could not be properly POS (Part-of-Speech) tagged by Stanford NLP (Natural Language Processing) library [8]. Most of these recipes are described in broken sentences. Some recipes on popular services such as Facebook did not contain description of real WoT apps and instead contained advertisements. We excluded these recipes. We also filtered out recipes that contained less than 3 words, as it would not bear sufficient information about a WoT app. We assume that users will issue requests that are similar to the refined IFTTT recipes.

**Fig 1:**
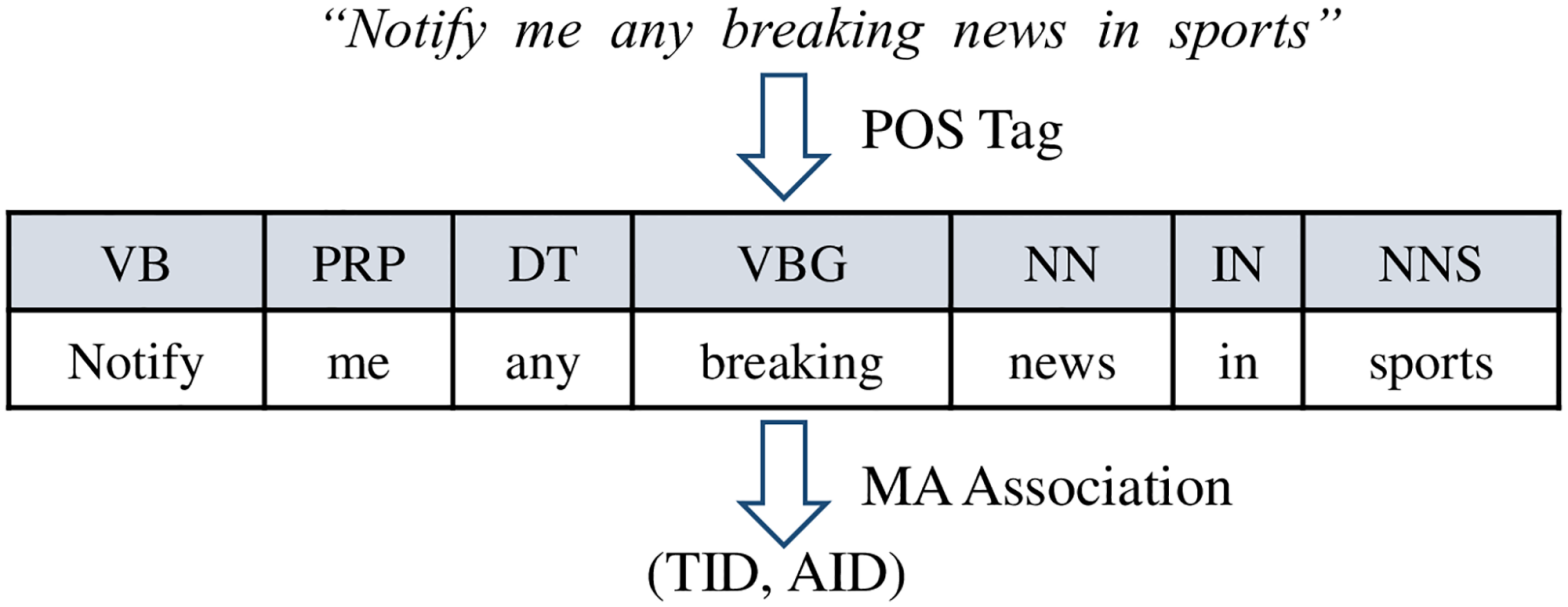
Training set generation for main act learning. A main act is a pair of the trigger function ID and the action function ID that were used in the WoT app. Our learning engine learns the association between the POS-tagged sequence and the main act.

Then, we POS-tag each tokens in every recipe using Stanford POS Tagger [8]. From these POS-tagged recipes, we generate two sets of training data, as shown in Fig 1. Each POS-tagged recipe in the first training set is associated with a pair of IDs of trigger and action functions that were actually composed together in the WoT app. We refer to this pair as a *main act*.

**Fig 2:**
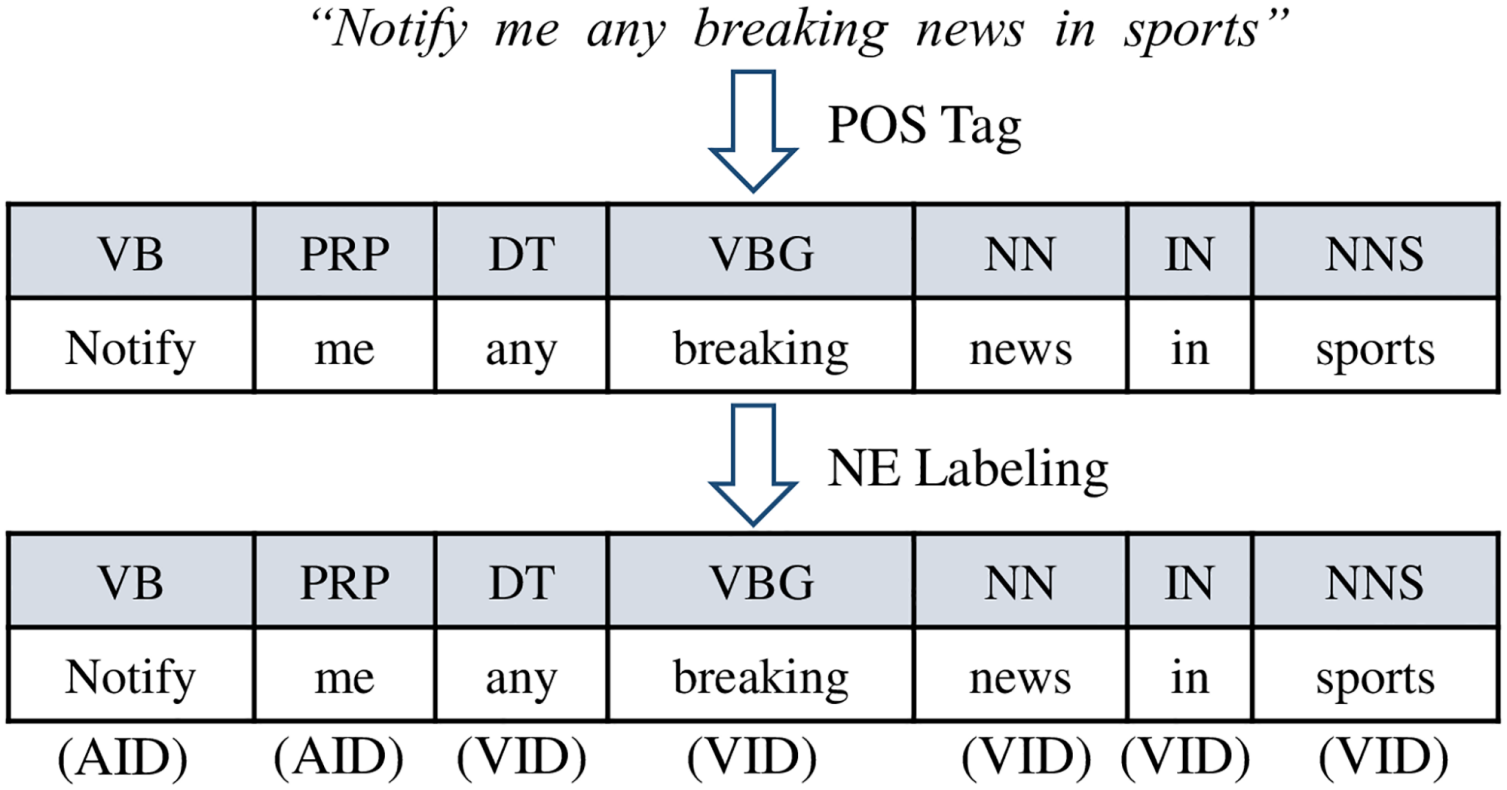
Training set generation for named entity recognition (named entity) learning. The named entity engine learns the association between the each POS-tagged token and the named entity which is either a trigger function ID or an action function ID.

Another method for generating a training set is illustrated in Fig 2. For each POS-tagged recipe, we can associate each POS-tagged token with either a trigger function ID or an action function ID. We refer to these labeled tokens as *named entities*. Such annotation is the feature that our learning engine has to learn. For instance, a recipe “Notify me any breaking news in sports” can be labeled as “VB/AID NN/AID DT/VID ADJ/VID NN/VID IN/VID SPORTS/VID” where AID is the ID of an action function and VID is the ID of a trigger function. We refer to the POS tag lists used in the Penn Treebank Project [9].

The ingredients (trigger and action functions) of the WoT app recipes are shown in the public page. However, there is no annotation that indicates which parts of the recipe refer to the description of the trigger or the action. We can manually annotate each token with a relevant named entity (a trigger function or an action function). However, this is an inefficient and time-consuming task. Instead, we handle such under-specified recipe metadata by leveraging an information retrieval technique as follows. We first store all recipes in ElasticSearch [10] and index them by trigger function IDs and action function IDs. Then, our system queries ElasticSearch to find the most relevant WoT function for each token in a recipe. ElasticSearch scores the relevancy between a token *t* and an index *i* by computing the cosine similarity between *t* and the TD/IDF-weighted frequency vector associated with *i*. Our system picks the index (either a trigger function ID or an action function ID) that returns the highest relevancy score and label the token to be associated with the index. Such association is what our named entity learning engine has to learn.

Our learning engine is based on modeling with conditional random fields (CRF) [6]. Specifically, we generate a first-order linear-chain CRF model in order to take advantage of discriminative modeling and sequence modeling as explained in [7].

The CRF-based modeling is formally defined as follows. First, partition function *Z*(*x*) is defined in Eq 1 where *x* is an input sequence of POS-tagged tokens in a recipe, and *y*′ is a sequence of named entity labels.

$$\begin{array}{c} Z\left(x\right)=\sum_{y^{\prime }}exp\left\{F\left(y^{\prime },x\right)\right\} \end{array}$$ (1)

Scoring function *F*(*y*, *x*) for a length-N sequence is defined in Eq 2 where Λ is a matrix that contains the transition scores between two adjacent output NE labels, and Ω is a matrix that contains the observation scores between a POS-tagged input token *x* and an output named entity label *y*^*i*^. *y*^0^ is a special starting state.

$$\begin{array}{c} F\left(y,x\right)=\sum_{i=1}^{N}\left(\Lambda_{y^{i},y^{i-1}}+\Omega_{y^{i},x^{i}}\right) \end{array}$$ (2)

Given an input sequence of POS-tagged tokens *x*, conditional probability of an output sequence, *y*, is computed according to Eq 3.

$$\begin{array}{c} P\left(y|x\right)=\frac{exp\{F(y,x)\}}{Z(X)} \end{array}$$ (3)

In the training phase, the negative log likelihood as shown in Eq 4 is optimized for all parameters *θ*. A naive approach is to iterate over all *L*^*N*^ possible output sequences, which is computationally infeasible. Instead, we use one of the quasi-Newton methods that approximate BFGS with limited memory [11, 12]. The gradient descent on Eq 4 is computed by a forward-backward algorithm that uses computation complexity of *O*(*NL*^2^) where *N* is the number of POS-tagged tokens and *L* is the number of named entity labels. However, the forward-backward algorithm has to be executed for each training instance that has different partition function *Z*(*x*). Hence, the complexity is *O*(*NL*^2^*TG*) where *T* is the size of the training set and *G* is the number of gradient computations [13].

$$\begin{array}{c} \underset{\theta }{\text{argmin}}\sum_{i=1}^{N}-F\left(y_{i},x_{i}\right)+log\left(Z\left(x_{i}\right)\right) \end{array}$$ (4)

In case of modeling for main act learning, we remove the transition score matrix from Eq 2 as shown in Eq 5. *y*^*i*^ is a pair of trigger function ID and a action function ID. Complexity of parameter optimization in this case is *O*(*EA*) where *E* is the number of trigger function IDs and *A* is the number of action function IDs.

$$\begin{array}{c} F\left(y,x\right)=\sum_{i=1}^{N}\left(\Omega_{y^{i},x^{i}}\right) \end{array}$$ (5)

We present an intuitive example of predicting a relevant named entity sequences given the learned transition score matrix Λ and the observation scores Ω matrix.

Suppose a recipe phrase is “alarm if”. This phrase can be annotated with POS tags as *x* = (*VB*, *IN*). Conditional probability *P*(*y*|*x*) given the named entity output sequence *y* as (*TID*_1_, *AID*_1_) is calculated as follows.

$P\left(y|x\right)=\frac{1}{Z(x)}exp\left\{\lambda_{AID_{1},Start}+\omega_{AID_{1},VB}+\lambda_{TID_{1},AID_{1}}+\omega_{TID_{1},IN}\right\}$, where λ_*j*,*k*_ is a transition score between output label *j* and *k*, and *ω*_*j*,*k*_ is an observation score between output label *j* and an input token *k*. Transition and observation scores can be retrieved from the learned Λ and Ω matrices, respectively. A main act and an output sequence of named entities with the highest conditional probability are selected for a test input sequence.

## Per-service learning and two-phase testing

In this section we present an improved framework that implements separate learning per trigger service and two-phase testing techniques.

**Fig 3:**
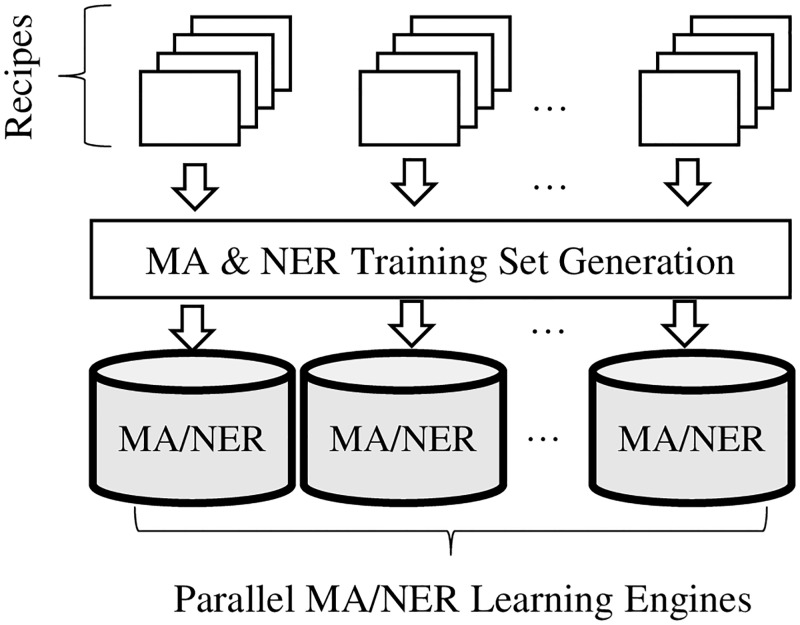
Generation of parallel learning engines. The CRF-based learning is conducted per trigger service.

In this framework, we first group IFTTT recipes by trigger services as shown in Fig 3. We label every recipe in each trigger service with a pair of trigger function ID and action function ID. As mentioned earlier this is to create a training set for main act learning. For modeling named entity sequences for recipes, we annotate every token with a relevant named entity. As presented in the previous section, we use the cosine similarity function in order to automatically select the most relevant trigger function ID or action function ID for every token in each recipe.

For each recipe group we separately conduct the linear-chain CRF modeling for supervise learning as we presented in the previous section. The number of output sequences the learning engine for each group has to consider for parameter estimation is orders of magnitude lower than the case where a single unified learning engine is trained. With this approach, we were able to scale up our learning procedures. Specifically, we were able to increase the total training data entries up to 270,000, which is 30 times more than the previous training data size. We had a total of 137 parallel engines that learned over 75,000 distinct features for learning main acts, which is 10 times more than what the previous learning engine learned. Also, our parallel engines learned over 5.5 million distinct features to learn named entities. This is 27 times more than what the previous learning engine could learn. For most of the parallel learning engines, it took only a few seconds to finish the training process. Therefore, it became feasible to reiterate the learning procedure whenever new recipe patterns are added to the training set. Also, the learning procedure can be embarrassingly-parallelized on multiple distributed machines.

**Fig 4:**
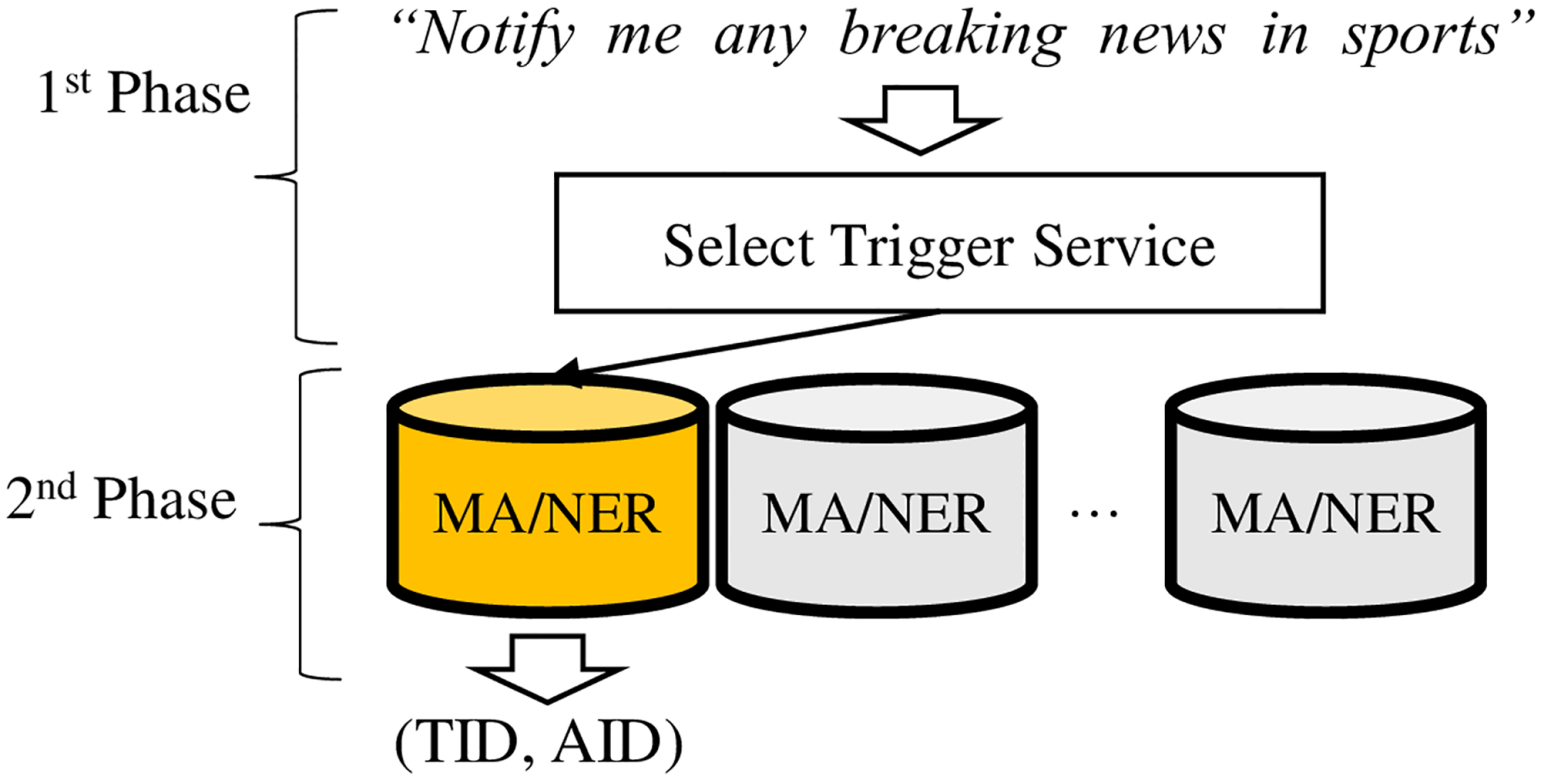
The two phase test approach. In the first phase, we use ElasticSearch to identify the best candidate that can yield triggers and actions relevant to a given WoT app request. In the second phase, the selected parallel learning engine conducts either main act classification or named entity recognition.

Given the parallel learning engines, the task of inferring a main act or a sequence of named entities is done in two phases as shown in Fig 4.

Among parallel learning engines, we select the one that is most likely to yield the relevant WoT functions for a given recipe query. Similar to the process of generating training data for the named entity learning, we first store documents of WoT app recipes and service descriptions into ElasticSearch and index them by trigger service IDs. ElasticSearch computes the similarity score between a recipe queries and all the indexed documents. We pick an index that yields the highest score according to the cosine similarity function. We identify the trigger service with the highest score. Then we retrieve the learning engine associated with the trigger service.

In the second phase, we POS-tag the given recipe query and classify its main act or recognize the named entity sequence through the learning associated with the relevant trigger service.

Through the decomposition of training sets and parallel learning, we anticipate the accuracy to be improved significantly. In the following section, we present the evaluation results.

## Results

We generated two sets of training data out of 270,000 publicly available recipes from IFTTT. Our system assigned a unique ID for every trigger and action we scrapped from these recipes. We also randomly sampled 80,000 recipes to generate a test set. We executed training and testing on an Ubuntu 14.04 server with Intel i5 3.2GHz CPU and 4GB of memory.

The average inference accuracy (*ψ*) of the engine is measured as follows.

$$\begin{array}{c} \psi =\frac{T+A}{R}*100 \end{array}$$ (6)

T is the total number of correctly identified trigger function IDs, A is the number of correctly identified action function IDs and R is the total number of recipes being tested. The maximum accuracy is 100.

The error rate (*ε*) is measured as follows.

$$\begin{array}{c} \varepsilon =100-\psi \end{array}$$ (7)

Table 1 shows the average accuracy of our new approach compared to the previous approach.

**Table 1:** Comparison of the basic and the new parallel learning approach in terms of main act and named entity learning accuracies.

	Basic	Parallel Learning
main act Learning	51.6	**78.4**
named entity Learning	32.8	**81.0**

The per-service named entity learning approach yielded the highest accuracy. It achieved 146% improvement over the basic named entity learning approach. The per-service main act learning approach made 52% improvement over the basic approach.

As shown in the table above, the new named entity learning approach outperformed the previous approach by 146%. In case of the main act learning, the accuracy increased by 52% compared to the basic main act learning approach. We observed the highest accuracy when the per-service named entity learning was conducted (81.0%). When compared to the best case, the new approach improved the accuracy by 56.9%. Under the new learning framework, the named entity learning approach showed higher accuracy than the main act learning approach. Named entity learning considers the transition scores in the parameter estimation while main act learning does not. Taking into account the transition between output labels is more beneficial for our new approach.

Despite the significant improvement in terms of accuracy, the error rates of the parallel named entity and main act testing are 19.0% and 21.6%, respectively. We attribute the non-negligible error rates to the inherently ambiguous expressions of the recipes that could have confused our learning engines. To quantify the ambiguity of the recipes, we define D_score_ and V_score_ in Eqs 8 and 10, respectively as follows.

$$\begin{array}{c} D_{\text{score}}=D-\sigma_{D} \end{array}$$ (8)

D is the number of unique recipes per given combination of trigger function ID and action function ID. *σ*_*D*_ is defined in Eq 9.

$$\begin{array}{c} \sqrt{\frac{\sum {\left(D_{\text{freq}}-\mu_{D}\right)}^{2}}{D}} \end{array}$$ (9)

D_freq_ is the frequency of each recipe in either a training set or a test set, and *μ*_*D*_ is the mean frequency of the recipes. *σ*_*D*_ is the standard deviation of frequencies of the unique recipes.

The lower the *σ*_*D*_ is, the more it contributes to the D_score_. A high D_score_ indicates that there are many diverse ways to express a request for a WoT app.

$$\begin{array}{c} V_{\text{score}}=V-\sigma_{V} \end{array}$$ (10)

V is the number of unique combination of trigger function ID and action function ID per recipe. *σ*_*V*_ is defined in Eq 11.

$$\begin{array}{c} \sqrt{\frac{\sum {\left(V_{\text{freq}}-\mu \right)}^{2}}{V}} \end{array}$$ (11)

V_freq_ is the frequency of each combination of trigger function ID and action function ID in either a training set or a test set, and *μ*_*V*_ is the mean frequency of the combinations.

Similar to *σ*_*D*_, *σ*_*V*_ is the standard deviation of frequencies of unique combinations. The lower the *σ*_*V*_ is, the more it contributes to the V_score_. A high V_score_ indicates that a recipe can refer to various trigger function ID and action function ID combinations.

**Fig 5:**
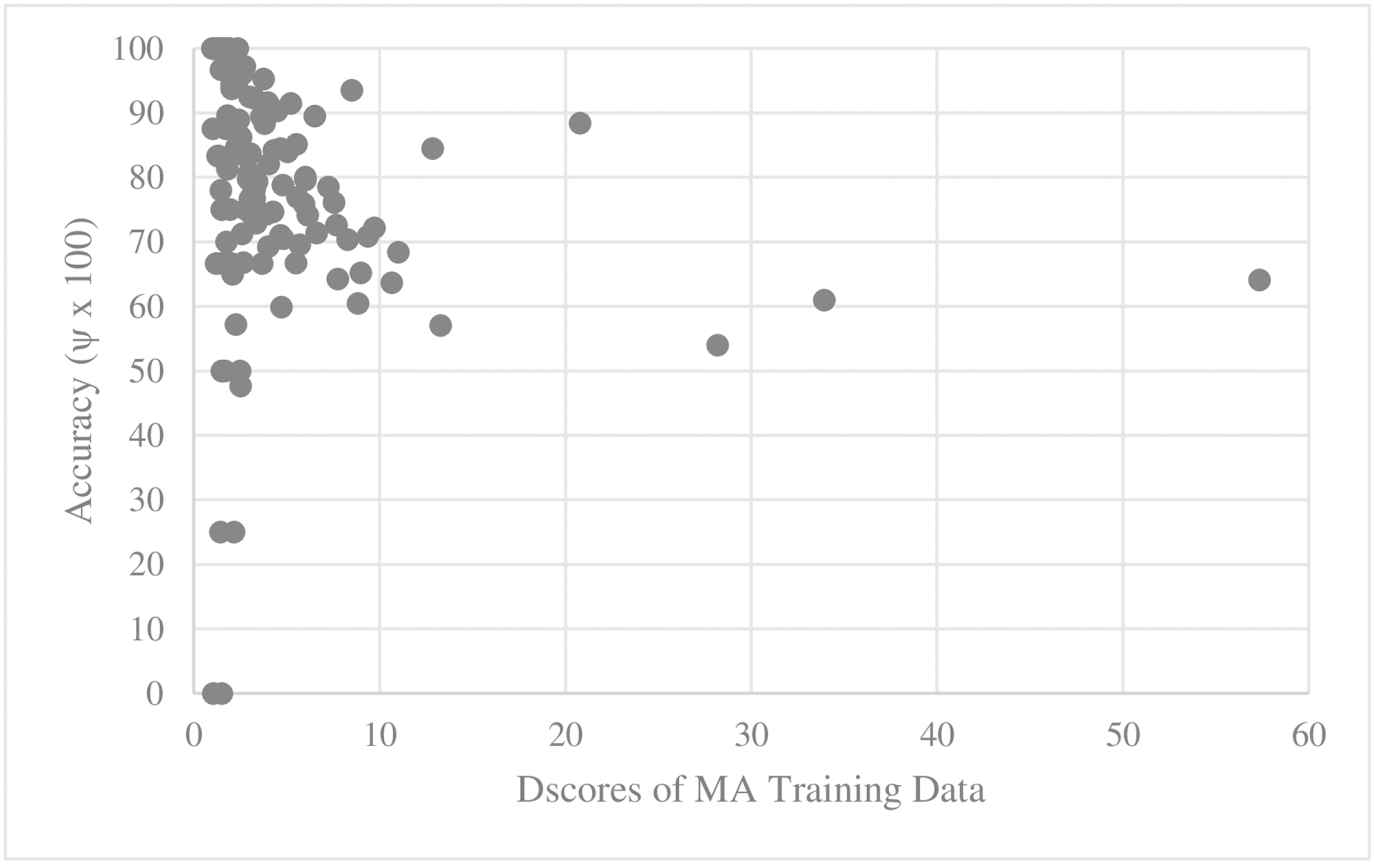
Main act learning accuracy against training data D_score_.

**Fig 6:**
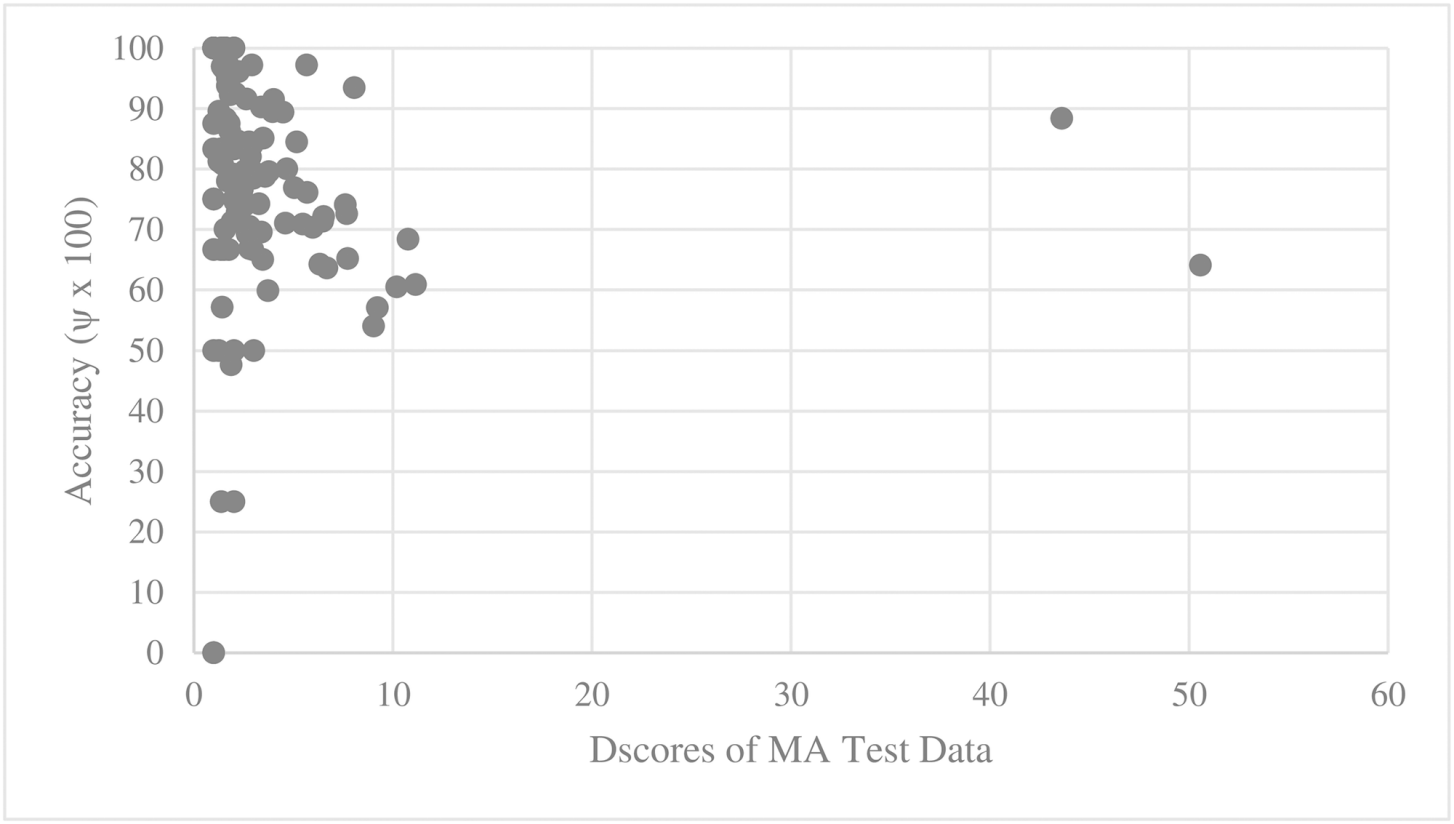
Main act learning accuracy against test set D_score_.

**Fig 7:**
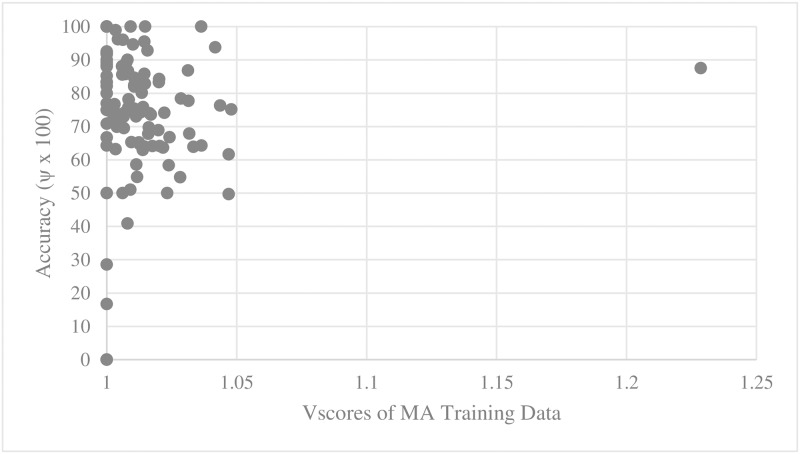
Main act learning accuracy against training data V_score_.

**Fig 8:**
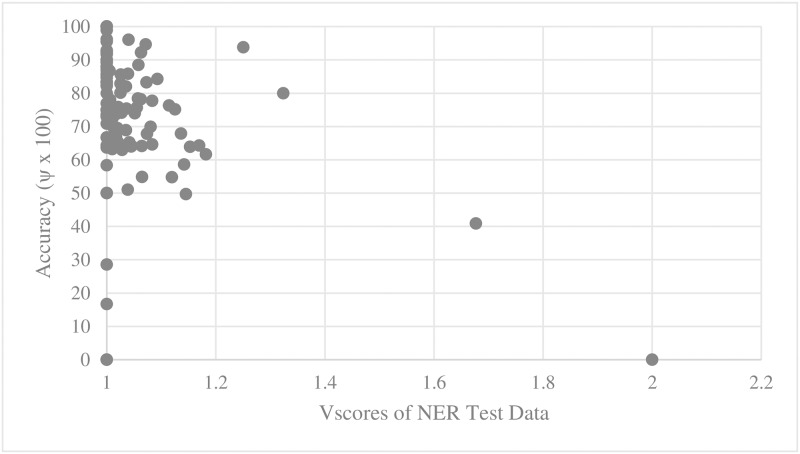
Main act learning accuracy against test set V_score_.

For every training set and test set for parallel main act and named entity learning engines, we computed the average D_score_ and V_score_. We then scatter-plotted average accuracy against D_score_ and V_score_. Figs 5, 6, 7 and 8 show the relationship between scores and the accuracies of main act learning.

**Fig 9:**
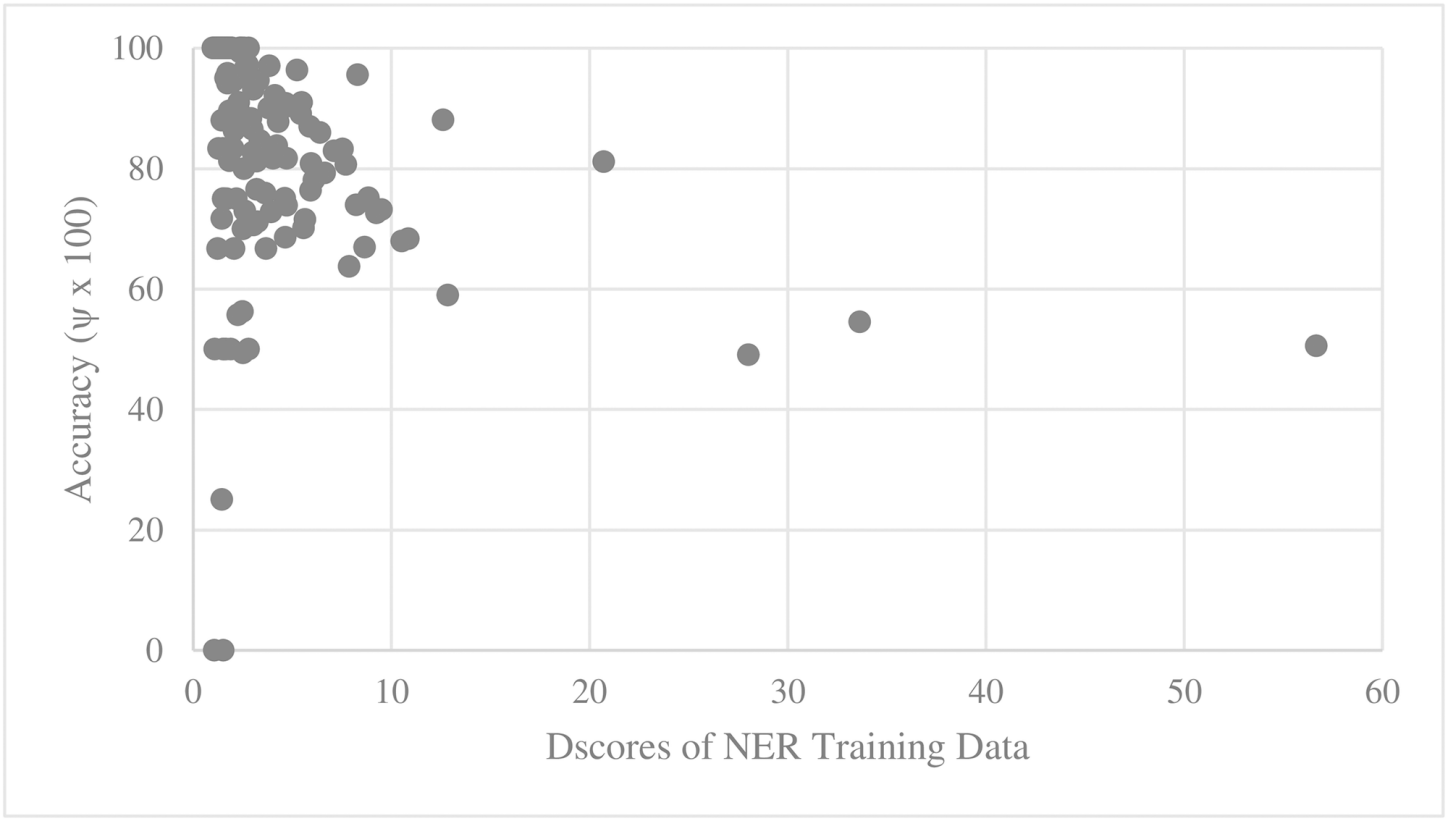
Named entity learning accuracy against training data D_score_.

**Fig 10:**
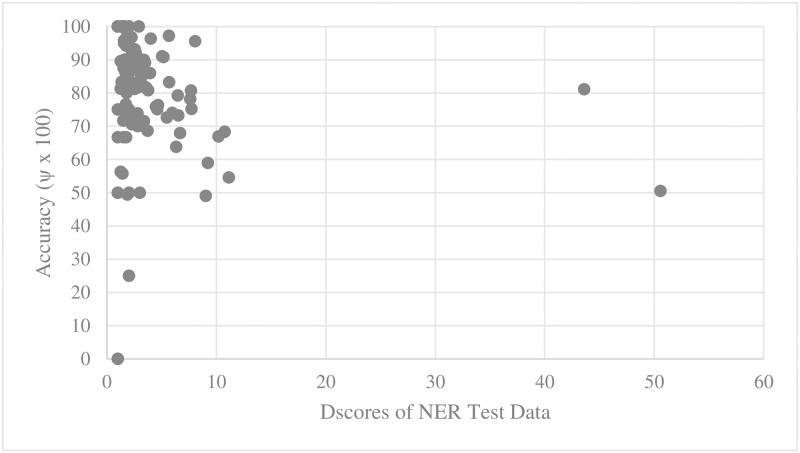
Named entity learning accuracy against test set D_score_.

**Fig 11:**
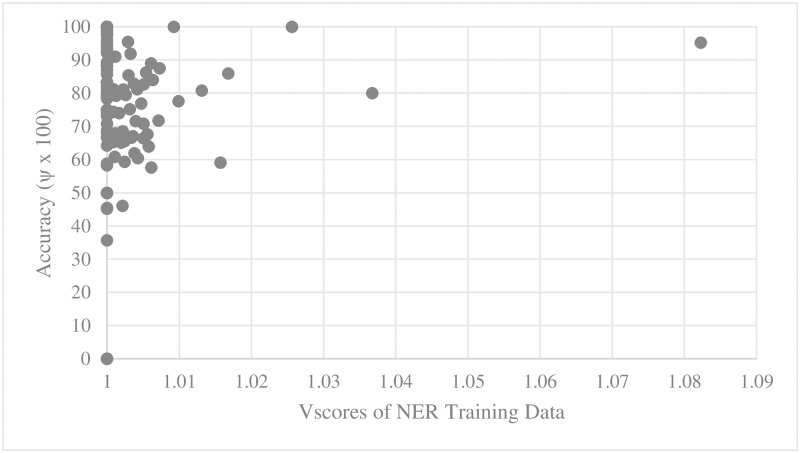
Named entity learning accuracy against training data V_score_.

**Fig 12:**
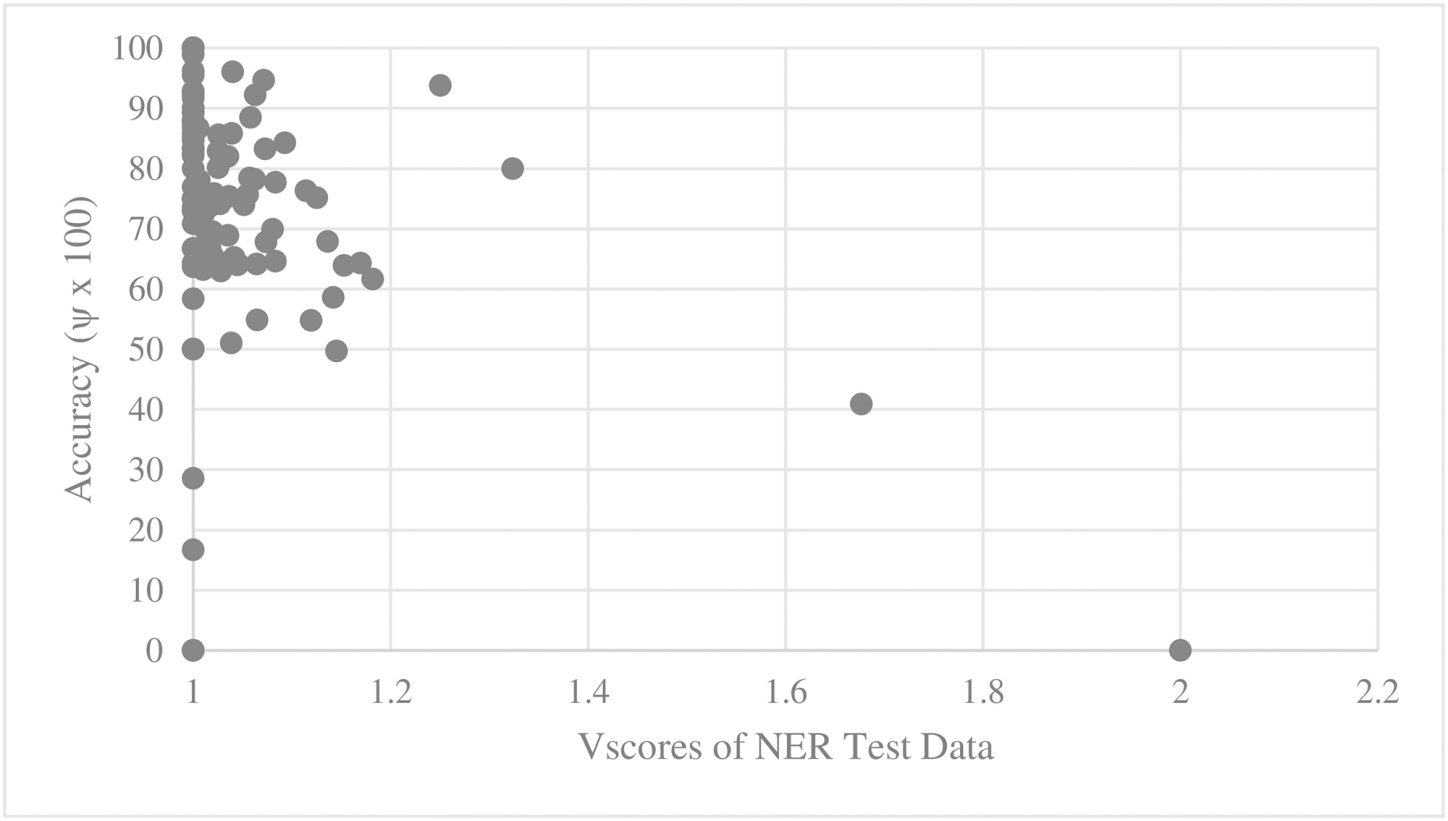
Named entity learning accuracy against test set V_score_.

Figs 9, 10, 11 and 12 show the trend of named entity learning accuracy in terms of D_score_ and V_score_.

The analysis result of correlation between the D_score_, the V_score_ and the accuracies is given in Table 1. Specifically, we computed Spearman’s rank correlation coefficient r [14]. As shown in Table 2, there was a significant negative relationship between D_score_, V_score_ and both the main act and named entity learning accuracies (with p-value < 0.0001). That is, the learning accuracy was high when D_score_ and V_score_ of both the training and test sets were low. This statistically shows that ambiguous expressions can confuse our learning engines.

**Table 2:** Spearman’s rank correlation coefficient (r) to assess the statistical relationship between D_score_, V_score_, the training set size and the learning accuracy.

	D_score_(*TE*)	D_score_(*TR*)	D_score_(*TE*) * D_score_(*TR*)	Training Set Size
main act Learning	0.821254224	0.847515728	0.847793418	0.402885639
named entity Learning	0.785341255	0.821968283	0.806816697	0.390793306
	V_score_(*TE*)	V_score_(*TR*)	V_score_(*TE*) * V_score_(*TR*)	Training Set Size
main act Learning	0.653002701	0.703239122	0.7234359778	0.375991178
named entity Learning	0.619526974	0.494039805	0.611542756	0.389870522

The coefficients were statistically significant with p-value < 0.0001 according to the Spearman test in R with degree of freedom (df) of 134 and 135, respectively for the analysis on D_score_ and V_score_. *TE* and *TR* stand for test set and training set, respectively.

Table 2 also shows that there was statistically positive relationship between the training set size and the learning accuracies (with p-value < 0.0001). The correlation coefficient r was less than 0.5.

**Fig 13:**
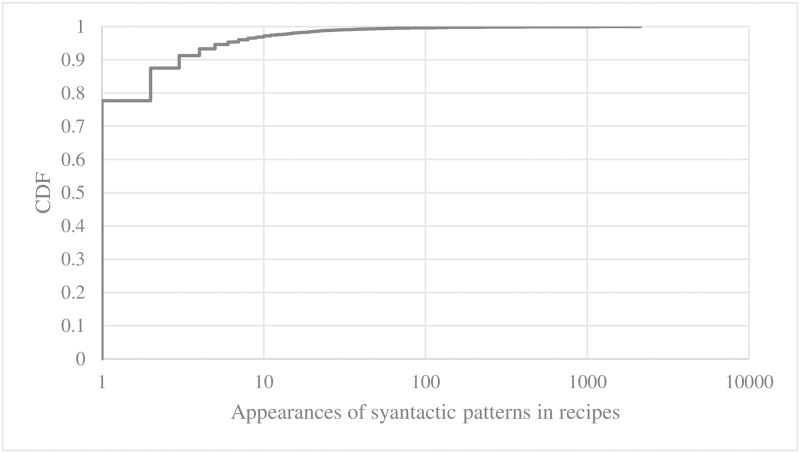
CDF of appearances of POS-tagged sequence patterns in recipes.

**Fig 14:**
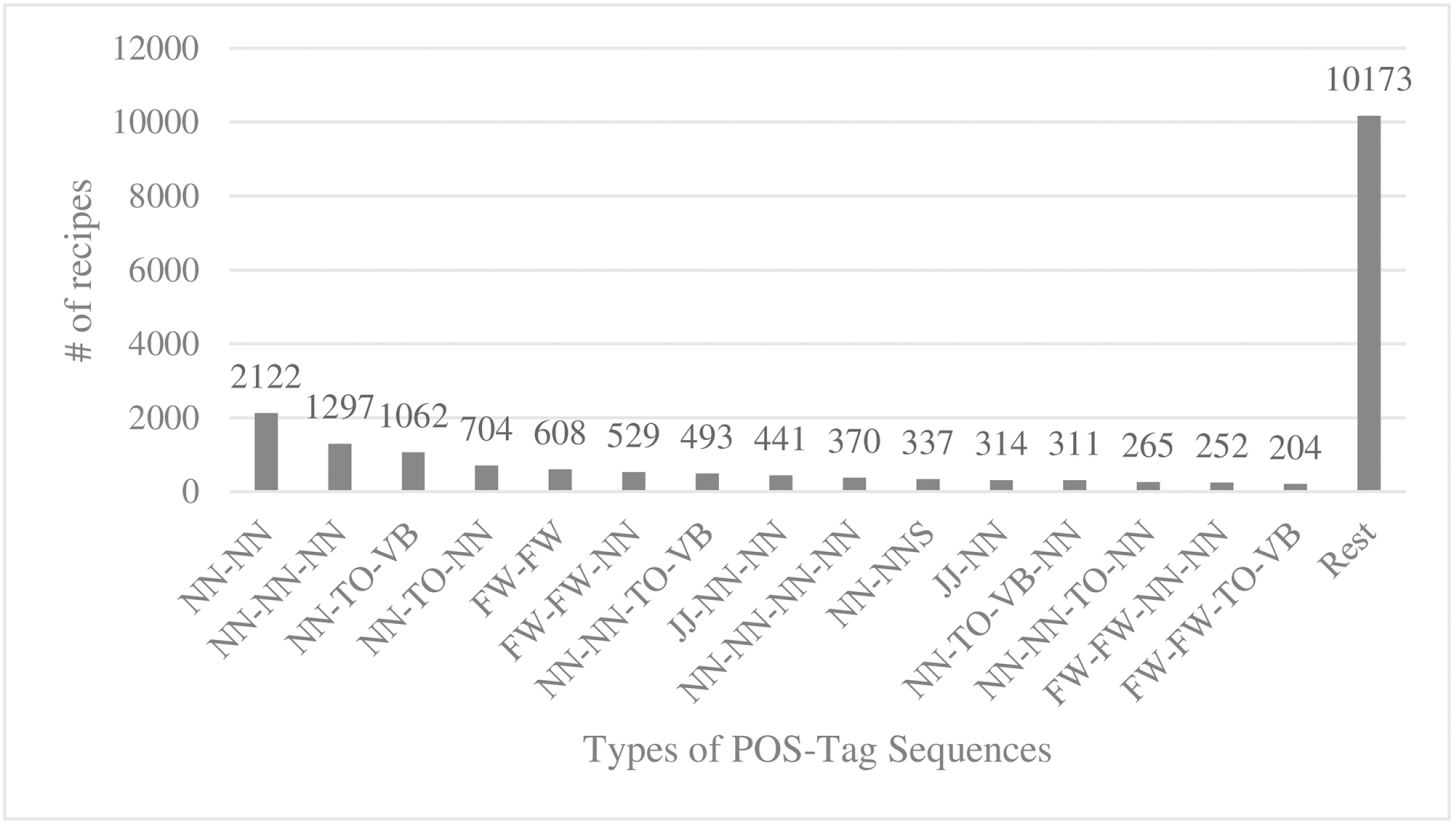
Top-15 pos-tagged sequence patterns in terms the frequency of appearance in recipes.

In the following, we look into the syntactic patterns of every public recipe we collected from IFTTT. In order to extract the syntactic patterns, we POS-tagged every recipe using the Stanford NLP library [8]. We identified 7,376 types of POS-tagged sequences. We also measured how frequently each POS-tagged sequence pattern appeared in recipe expressions. We plotted CDF of appearances of POS-tagged sequence patterns in Fig 13. In Fig 14, we show top-15 pos-tagged sequence patterns in terms the frequency of appearance in recipes.

We observed that 76% of the entire recipes followed one of the top 20% of the POS-tagged sequence patterns. 24% of the recipes followed one of the bottom 80% of the POS-tagged sequence patterns. 90% of the POS-tagged sequence patterns appeared in less than 3 recipes. In short, the appearance of the POS-tagged sequence patterns in recipes were highly skewed while the types of POS-tagged sequences varied significantly.

**Fig 15:**
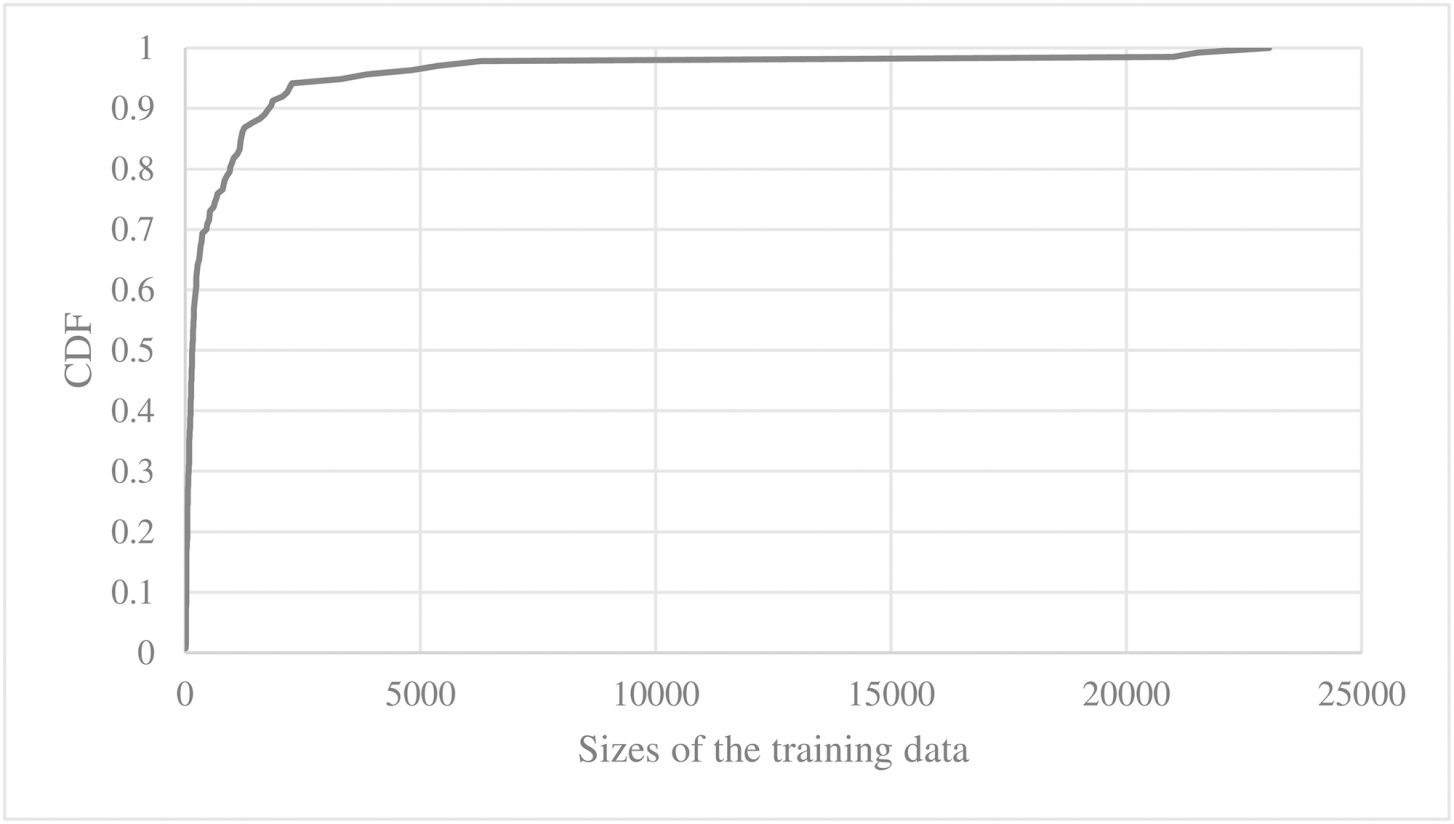
CDF of sizes of training data for the parallel engines.

We can see that the learning was done mostly on the top 20% of the syntactic patterns, and this biased learning could have caused an overfitting problem. We show the unbalanced learning phenomenon on a different angle. In Fig 15, we show CDF of training data sizes of trigger services. While 90% of the parallel learning engines learned less than 1,482 training entries, each of top-3 trigger services such as Feed, Twitter and Instagram learned more than 20,000 training entries. Average accuracies of the learning engines that were trained with the data set from these top-3 trigger services were only 59.7% which was less than the accuracy of the entire learning engines.

Overcoming such an unbalanced learning is challenging since the popularity of particular syntactic patterns and services among the real users are out of our control. We can re-configure the training set to have equal number of training entries per syntactic pattern or service. However, this would significantly reduce the overall training data size. When we limited training entry to 1 for every syntactic pattern (POS-tagged sequence), the accuracy dropped by 82%.

We can summarize the key observations from the empirical evaluation as follows:

1. Learning accuracy improved by at most 146% compared to the basic approach.
2. Ambiguity of the recipe expressions were quantified and we showed statistically that it had negative effect on the learning accuracies.
3. Training data were skewed towards some syntactic patterns and trigger services.

## Discussion

In this section, we mainly discuss future research directions for improving the learning accuracies further.

Recall that we employed ElasticSearch to classify recipes to a relevant trigger service. However, it turns out that ElasticSearch correctly classified only 90% of the recipes that were used for the training set and the test set. This limited the accurate identification of the actual trigger and action IDs. We also relied on ElasticSearch to label each token with either a trigger function ID or an action function ID for named entity learning. This also limited the named entity accuracy. A manual yet precise classification and labeling work can improve the learning accuracies significantly. However, employing the costly human resource to conduct the precise annotation work is impractical especially when the corpus for the WoT function descriptions is expected to grow continuously.

We can consider adopting some of the latest NLP techniques based on deep learning to extract the semantics of recipes more effectively [15, 16]. This may help us increase the accuracy in identifying which parts of a given recipe corresponds to the description of a trigger or an action. However, we are keen to our findings that some of the patterns were disproportionately frequent, while the number of tokens used in the patterns were mostly less than 10. We can consider defining rules for the few frequently appearing patterns manually as oppose to solely relying on machine learning techniques. For instance (Facebook/NN, to/TO, Gmail/NN) is one of the most frequent POS-tagged patterns. We can derive a deterministic rule that the former NN refers to the trigger function and the latter NN refers to the action function.

Another line of future work is to develop a way to return top-k relevant pairs of trigger and action IDs as oppose to returning just one pair. Presenting multiple candidates would give user more options and increase the likelihood of finding satisfactory WoT apps. This requires implementing a dialog management system to suggest candidate answers to the users and ask for more elaboration. It would be an interesting challenge to find a way to minimize the number of clarification questions to the user, since it significantly impacts the usability.

## Related work

We put our work in the context of voice-based personal assistant services, natural language processing and semantic mashups.

There are a number of voice-based personal assistant apps such as Siri [17], Cortana [18], Alexa [19] and Google Assistant [20]. These state-of-the-art apps rely heavily on knowledge bases to answer user inquiries. However, none of these apps work in concert with WoT ecosystems at a scale close to IFTTT or Zapier. There are a few domain-specific technologies for controlling workflows over voice in clinical environment [21, 22]. LingoLogic is a menu-based natural language interface to query semantic web [23]. To the best of our knowledge, we have pioneered the research on how to process users request to compose event-drive applications from hundreds of WoT services that offer thousands of functionalities. In [4], we actually demonstrated a prototype that has a voice interface to the IFTTT platform. This work has motivated us to study the ways to effectively handle recipe requests issued in natural language.

We can consider adopting latest research work on natural language understanding, especially the ones that are based on sequence-to-sequence modeling. In [15], a pair of recurrent neural networks were used to build a smart email reply system that can capture sentences that are semantically similar to each other. In [16], skip-thought vectors were used to represent off-the-shelf sentence representations. This work was evaluated on various natural language understanding tasks such as recognizing semantic relatedness and classifying question types. These techniques can be helpful in identifying a trigger service relevant to a given recipe. However, there is still a critical prerequisite of generating a training set that explicitly identifies the portion of recipes that correspond to an trigger description or an action description.

Guinard et al. introduced a resource-oriented approach of wrapping physical sensors with RESTful interface, so that they can interact with each other through web [24]. However, to ensure that heterogeneous WoT entities exchange services and data unambiguously, *semantic interoperability* should be satisfied first [25, 26]. In order to guarantee semantic interoperability, the research community has studied methods to semantically annotate the data and services offered by WoT entities. Heflin et al. [27] presented a semantic annotation language that is more effective than XML. McIraith et al. worked on standardizing the semantic annotation of web services [28]. W3C later extended the discussion of the semantic annotation for describing semantic sensor networks [29]. In the context of Internet of Things, SmartThings platform implemented *Physical Graph* that is used for automatically discovering the capabilities of home automation devices [30]. However, we had to resort to search engines with inverted indexing (e.g., ElasticSearch) in order to match recipes against unstructured WoT service information. We can study further to see whether enriched semantic annotation of the training data, test data and WoT services enhances the learning accuracies.

## Conclusion

In this paper, we explained the implementation of the framework that enables per-service learning and two-phase testing for automatically recognizing the necessary WoT functions for a given user request issued in natural language. Using this approach, the accuracy increased by 146% and 51% for named entity learning and main act learning, respectively. Since we have separate learning engines for each trigger service, parallelizing the learning process became possible. Thanks to the efficient training per service, it became more feasible to conduct incremental learning whenever new recipe patterns are introduced.

More state-of-the-art supervised learning techniques can be considered besides the CRF-based learning in the future work. However, we also think that it is worth to consider adding human engineers into the loop to manually define parsing rules for the diverse but disproportionately frequent syntactic patterns observed in the recipe expressions. Finally, we plan to extend the work to generate multiple candidate recipes to the users in order to increase the likelihood finding the most satisfactory WoT functions.
